# Riboflavin transporter deficiency in young adults unmasked by dietary changes

**DOI:** 10.1002/jmd2.12427

**Published:** 2024-06-27

**Authors:** Bregje Jaeger, Mirjam Langeveld, Robert Brunkhorst, Felix Distelmaier, Ana Pop, Nicole I. Wolf, Annet M. Bosch

**Affiliations:** ^1^ Department of Child Neurology Emma Children's Hospital, Amsterdam University Medical Centers Amsterdam The Netherlands; ^2^ Department of Endocrinology and Metabolism Amsterdam University Medical Centers Amsterdam The Netherlands; ^3^ Department of Neurology Aachen University Medical Center Aachen Germany; ^4^ Department of General Pediatrics, Neonatology and Pediatric Cardiology University Children's Hospital, Heinrich Heine University Düsseldorf Germany; ^5^ Laboratory of Genetic Metabolic Diseases, Gastroenterology, Endocrinology & Metabolism Amsterdam University Medical Centers Amsterdam The Netherlands; ^6^ Department of Child Neurology Emma Children's Hospital, and Amsterdam Neuroscience, Cellular & Molecular Mechanisms, Vrije Universiteit Amsterdam The Netherlands; ^7^ Department of Pediatrics, Division of Metabolic Disorders Emma Children's Hospital, Gastroenterology, Endocrinology & Metabolism, Amsterdam University Medical Centers Amsterdam The Netherlands

**Keywords:** neurodegenerative disease, riboflavin transporter deficiency, vitamin B2, young adult

## Abstract

Riboflavin transporter deficiency (RTD) is a genetic disorder of reduced riboflavin (vitamin B2) uptake that causes progressive, multifocal neurological dysfunction. Most patients present in early childhood; if patients present later in life, symptoms usually develop more gradually. We report three previously healthy young adults, who developed rapidly progressive neurological symptoms after decreasing dietary intake of meat and dairy. After a diagnostic odyssey, the diagnosis of a riboflavin transporter deficiency was made. Treatment with high dose oral riboflavin (20–40 mg/kg/day) partially reversed symptoms. This case series highlights that reduced riboflavin intake as a result of dietary changes can unmask RTD at a later age. We emphasize the importance of early recognition of this progressive and potentially lethal disease and show that timely treatment with high dose riboflavin is highly effective.

**Synopsis**

A decrease in dietary riboflavin intake can unmask a previously unknown riboflavin transporter deficiency in adulthood. Prompt treatment with high dose oral riboflavin is of vital importance.

## 1 INTRODUCTION

Riboflavin transporter deficiency (RTD, OMIM 614707), formerly known as Brown‐Vialetto‐Van Laere (BVVL) or Fazio Londe syndrome (OMIM 211500) is an inborn error of metabolism caused by variants in *SLC52A2* or *SLC52A3*, encoding the human riboflavin transporters RFVT2 and RFVT3, respectively. More than a 100 genetically confirmed cases of riboflavin transporter deficiency have been reported to date.^1^

Onset of symptoms may occur from early infancy until adulthood. Presentation at a young age tends to be rapidly progressive, with a fatal outcome when untreated. When symptoms start after early childhood, disease course is more gradual. Presenting signs and symptoms of RTD are loss of hearing and vision, facial weakness and difficulty speaking and swallowing as a result of a multiple cranial neuropathy. Muscle weakness and a sensory ataxia are caused by a peripheral neuropathy. Paralysis of the diaphragm can lead to respiratory insufficiency. Timely treatment with oral riboflavin halts disease progression and is lifesaving.^1^

We describe three previously healthy young adults who developed symptoms of RTD after a switch to a diet with less meat and dairy.

## 2 CASE REPORTS

For all cases, details on neurological examination and additional testing are given in Table 1.

**TABLE 1:** Clinical, biochemical, and radiological data of case 1–3.

	Case 1	Case 2	Case 3
*General information*			
Age at onset (years)	21	22	17
Age at diagnosis (years)	22	23	17
Family history	−	−	−
Time from diet change to symptoms (months)	12	Several	Several
Time between symptoms and start of B2 (months)	7	11	6
*Clinical symptoms*			
Hearing loss	−	+	+
Vision loss	−	+	−
Bulbar weakness	++	+	+
Muscle weakness	++	+	−
Sensory symptoms	+	−	−
Respiratory symptoms	−	−	−
Neurological examination			
Cranial nerves	Nystagmus, bilateral facial weakness, tongue atrophy with fasciculations, hoarse, high pitched voice	Vision 0.3 in both eyes, bilateral facial weakness, facial numbness, fasciculations tongue, hoarse, high pitched voice	Bilateral facial weakness, unilateral ptosis, dysarthria
Motor function	Severe distal, mild proximal weakness in legs. Increased muscle tone in legs. Bilateral atrophy of tibialis anterior	Mild distal weakness in arms, severe distal weakness in legs	No obvious impairment
Reflexes	Brisk in arms, pathologically increased in legs, extensor plantar responses	Normal except for decreased Achilles tendon reflexes	Normal
Coordination	Intact	Intact	Intact
Sensory function	Sensory ataxia	Intact	Intact
Biochemical abnormalities (ref in plasma)			
Acylcarnitine profile	N.A.	*C8‐carnitine*: 0.53 (0.04–0.22) *C10:1‐carnitine*: 0.25 (0.04–0.22) *C10‐carnitine*: 0.64 (0.04–0.30) *C12‐carnitine*: 0.16 (0.04–0.14) *C14:1‐carnitine*: 0.22 (0.02–0.18)	N.A.
FAD (nmol/l)	N.A.	46.2 (46–114)	N.A.
FMN (nmol/l)	N.A.	1.6 (0.8–21.4)	N.A.
Riboflavin (nmol/l)	N.A.	1.2 (3.9–49)	N.A.
Imaging			
MRI brain	Bilateral T2 hyperintensity of corticospinal tracts	Bilateral enhancement of the trigeminal, facial and acoustic nerves	Contrast enhancement of both facial nerves and the right vestibulocochlear nerve
MRI spinal cord	Enhancement of anterior nerve roots of cauda equina	Enhancement of cauda equina nerve roots	Normal
CSF			
Leucocytes (ref 1–10E6/L)	1	1	1
Protein (ref 0.24–0.49 g/L)	1.1	0.7	0.5

Abbreviation: N.A., not available.

All patients provided written informed consent for publication.

### 2.1 Case 1

A 21‐year‐old female with an unremarkable medical history switched to a diet with considerably less meat and no dairy products. One year later, she developed a tonsillitis, followed by facial weakness and leg weakness.

Initial workup revealed a cytoalbuminologic dissociation in CSF. EMG demonstrated acute denervation in the legs and facial muscles. Intravenous immunoglobulines were given for presumed, atypical Guillain–Barré syndrome. Over the next 2 months, she developed difficulties speaking and swallowing and was unable to walk. Percutaneous gastric tube feeding (PEG) and a urine‐catheter were placed. Neurological examination revealed multifocal deficits.

MRI of brain and spinal cord showed a bilateral, symmetric, hyperintense T2 signal in the corticospinal tracts and enhancement of the anterior cauda equina radices. Genetic analyses were performed since the disease course was atypical for Guillain‐Barré syndrome.

Exome sequencing revealed compound heterozygous variants in *SLC52A3* (Table 2), consistent with a riboflavin transporter deficiency type 3. She started oral treatment with riboflavin (20 mg/kg/day).

**TABLE 2:** Variants identified in *SLC52A3* (Reference sequence: NM_001370085.1).

Case no.	Nucleotide change	Predicted change	Allele frequency (gnomAD v.2.1.1, % of control alleles)	Pathogenicity according to ACMG standards and guidelines^a^	Variant reference
1	c.742del	p.(Trp248Glyfs*41)	N.D.	Pathogenic (PVS1, PM2, PP5)	This report
1	c.796C > T	p.(Arg266Trp)	0.002	*Likely pathogenic* (PS1, PM2, PP3)	Ciccolella et al., 2012
2	c.374C > A	p.(Thr125Asn)	0.003	*Likely pathogenic* (PS1, PM2, PP3)	Manole et al., 2017; Chaya et al., 2018
2	c.1381G > T	p.(Asp461Tyr)	0.04	*Likely pathogenic* (PS1, PM2, PP3)	Bashford et al., 2017
3	c.917 T > C	p.(Leu306Pro)	N.D.	Uncertain significance (PM2, PP3)	This report
3	c.944A > G	p.(Asn315Ser)	N.D.	Uncertain significance (PM2, PP3)	This report

Abbreviations: N.D., not detected in gnomAD; PVS1, pathogenic very strong, null variant (frameshift); PM2, pathogenic moderate, absent from controls (or at extremely low frequency); PP5, pathogenic supporting, reputable source recently reports variant as pathogenic but the evidence is not available to the laboratory to perform an independent evaluation; PS1, pathogenic strong, same amino acid change as a previously established pathogenic variant; PP3, pathogenic supporting, multiple lines of computational evidence support a deleterious effect on the gene or gene product (conservation, evolutionary, splicing impact, etc.).

^a^ Richards et al., 2015. Genet Med.

A year later, swallowing and urination had returned to normal. Facial strength and speech had improved. Currently, she is able to walk 500 m with a walking aid.

Her asymptomatic sister carried the same variants. She followed a vegetarian, non‐vegan diet and had a normal acylcarnitine profile and normal plasma values of riboflavin.

### 2.2 Case 2

A 23‐year‐old male without previous medical problems reduced his intake of dairy products and green vegetables. A few months later, he developed facial weakness, hearing loss and difficulties speaking and swallowing. Six months later he developed a rapidly progressive, bilateral foot drop, weakness in his hands and loss of vision (0.3 in both eyes). Because of loss of taste and dysphagia, he lost 25 kg in weight. On examination there was dysfunction of multiple cranial nerves and distal muscle weakness in arms and legs.

MRI showed bilateral enhancement of the trigeminal, facial and acoustic nerves, and enhancement of the cauda equina radices. CSF analysis revealed an elevated protein level without leucocytes. Nerve conduction studies were consistent with a sensorimotor axonal polyneuropathy. Electromyography showed signs of muscle denervation.

IVIG and subsequently methylprednisolone pulse therapy were ineffective.

Metabolic screening revealed an abnormal acylcarnitine profile and a low level of flavin mononucleotide (FMN) and riboflavin.

Genetic testing confirmed a riboflavin transporter deficiency due to compound heterozygosity for two variants in *SLC52A3*. Both parents were carrier of one variant. A year after starting riboflavin treatment (26 mg/kg/day), speech, and swallowing had returned to normal. In the ensuing years, muscle weakness in the legs improved. Walking distance with orthopedic shoes is unimpaired. Hearing and vision did not improve. He successfully received cochlear implants.

### 2.3 Case 3

A 17‐year‐old girl with an unremarkable history developed hearing difficulties within several months after switching to a vegetarian diet and reducing her intake of dairy products. Subsequently, she developed facial weakness and a change in voice. Cranial MRI demonstrated contrast enhancement of both facial nerves and the right vestibulocochlear nerve. CSF showed an increased serum versus CSF IgG‐index. A trial with corticosteroids and IVIG showed no effect. Exome sequencing revealed compound heterozygous variants in *SLC52A3* confirming the diagnosis of riboflavin transporter deficiency type 3. Symptoms gradually improved after start of oral riboflavin treatment (26 mg/kg/day, increased to 40 mg/kg/day).

## 3 DISCUSSION

We report on three young adults with rapidly progressive and multifocal neurological deficits due to a riboflavin transporter deficiency, unmasked by a change in diet, resulting in reduced intake of riboflavin.

Riboflavin is a water‐soluble vitamin that comes from dairy products, meat, fish and green vegetables. Riboflavin is not stored in the body, humans are fully dependent on dietary intake. The daily recommended intake of riboflavin for adults is 1.6 mg/day.^2^ Riboflavin content of milk (150 mL) is 0.27 mg, meat (75 g) 0.05 mg, and cooked vegetables (50 g) 0.04 mg, indicating that removal of dairy products from the diet significantly affects riboflavin intake.^3^ Furthermore, urinary riboflavin excretion has been reported to increase during infection.^4^

Riboflavin uptake takes place through three riboflavin transporters. Pathogenic, biallelic variants in *SLC52A2* and *SLC52A3* cause riboflavin transporter deficiency type 2 and 3.^5^ So far, no clinical phenotype caused by biallelic variants in *SLC52A1* coding for riboflavin transporter 1 has been reported. However in two newborns with clinical symptoms of riboflavin deficiency, the mothers harbored heterozygous *SLC52A1* variants (OMIM 615026).^6^, ^7^

There is limited insight regarding a genotype–phenotype correlation in RTD. However it is remarkable that the c.1381G > T variant in case 2 has been previously reported in compound heterozygosity in a 35‐year old patient with a subacute onset of RTD.^8^ In addition, the second variant in case 2 (c.374C > A) was reported in compound heterozygous form in a patient who first developed symptoms of RTD at the age of 10, but also in a boy with symptom onset at 10 weeks of age.^9^, ^10^

One of the variants in case 1 (c.796 C > T) has been reported in a homozygous state in a 9‐year‐old patient with subacute onset of RTD symptoms.^11^

Riboflavin is the precursor of flavin mononucleotide (FMN) and flavin adenine dinucleotide (FAD), cofactors for enzymes called flavoproteins, which play an essential role in fat, carbohydrate and protein metabolism.^12^ As flavines play an important role in mitochondrial function, mitochondrial dysfunction might contribute to neurodegeneration in RTD.^1^, ^10^

Mean age of presentation in a review of 70 genetically confirmed RTD 2 and 3 cases was 4.1 years (range 0.25–27.0 years; SD 5.0). In untreated patients presenting before age 4 years, the mean time span between presentation and death was 0.8 years.^13^

Presenting symptoms of RTD consist of subacute onset of cranial nerve deficits, muscle weakness and sensory loss. Children may develop respiratory insufficiency due to paralysis of the diaphragm. The majority of patients have striking bulbar weakness and sensorineural hearing loss. Vision loss due to optic atrophy, disorders of ocular motility and facial palsy were also reported.^1^ Muscle weakness is often accompanied by pronounced atrophy, predominantly in distal limbs, shoulder girdle and axial muscles. Vibration and position sense is more affected than loss of sensation for pain and touch, giving rise to a sensory ataxia.^14^

The cases presented here illustrate that diagnosing RTD can be challenging given its overlapping features with acute inflammatory neuropathies like Guillain‐Barré syndrome. Protein level in CSF in patients with RTD can be elevated just like in Guillain‐Barré syndrome. The etiology of the cytoalbuminologic dissociation in RTD is yet unclear. In some patients even a partial response to IVIG has been described.^15^, ^16^, ^17^ However, loss of vision and hearing, affected ocular motility and the presence of signs indicating upper motor neuron involvement, do not fit with typical Guillain‐Barré syndrome.

Neurophysiological findings in RTD are consistent with a sensorimotor axonal neuropathy.^18^ Rare post‐mortem reports describe findings of degeneration with neuronal loss and gliosis in lower cranial nerve nuclei (VII–XII), depletion of neurons in anterior horn cells and atrophy of spinocerebellar and spinothalamic tracts, along with an axonal neuropathy in peripheral nerves.^10^, ^18^, ^19^

Visual problems and a sensory ataxia seem to be more frequent in patients with RTD 2. In RTD 3, bulbar symptoms tend to occur earlier in the disease course compared to RTD 2. Late onset RTD has been documented only with RTD 3 variants.^1^, ^13^

In approximately 50% of RTD patients low plasma flavin levels and abnormal “multiple acyl‐CoA dehydrogenase deficiency (MADD) – like” plasma acylcarnitine profiles are found, indicative of an impairment in the metabolism of fatty acids by mitochondrial β‐oxidation.^1^, ^13^ Results of urine organic acid analysis are normal in half of RTD cases. Ethyl‐malonic aciduria suggestive of impairments in fatty‐acid, methionine and/or isoleucine oxidation is the most common abnormality noted. These biochemical abnormalities can raise suspicion of RTD. However, the gold standard in diagnosing RTD is identification of causative genetic variants in *SLC52A2* and *SLC52A3*.

Remarkably, the time between the change in diet and the display of symptoms in these patients varied from several up to even 12 months. Hypothetically this could be explained by residual riboflavin transporter function, leading to just sufficient riboflavin uptake when adequate intake is guaranteed, preventing symptom development.

The asymptomatic sister of case one was also compound heterozygous for both familial SLC52A3 variants and remained symptom free. She was vegetarian but did not reduce dairy content of her diet. In case one, possibly an increased loss of vitamin B2 due to an infection, upon a greatly reduced intake has set a cascade in motion that led to the development of serious symptoms.

Treatment with oral riboflavin (20–40 mg/kg/day in 3–4 doses) leads to gradual clinical improvement or stabilization of symptoms.^13^ Clinical improvement in these patients occurred within days to months after start of treatment and corresponds with what has been described in other reported patients.^14^, ^18^, ^20^, ^21^ When RTD is suspected, riboflavin treatment must be started immediately. Earlier start of treatment is associated with a better outcome.^13^

In a time where plant‐based diets are gaining ground, it is paramount that physicians realize that dietary changes can unmask symptoms of riboflavin transporter deficiency.^22^ As riboflavin is easily available and oral riboflavin treatment is highly effective, early recognition of this new RTD phenotype and prompt diagnosis and treatment are essential to prevent death or long‐term severe disability.

## AUTHOR CONTRIBUTIONS

Bregje Jaeger, Mirjam Langeveld, Robert Brunkhorst, Felix Distelmaier, Ana Pop, Nicole I. Wolf, and Annet M. Bosch: Drafting/revision of the article for content, including medical writing for content.

## FUNDING INFORMATION

The authors report no targeted funding.

## CONFLICT OF INTEREST STATEMENT

The authors declare no conflicts of interest.

## ETHICS STATEMENT

All procedures followed were in accordance with the ethical standards of the responsible committee on human experimentation (institutional and national) and with the Helsinki Declaration of 1975, as revised in 2000 (5). Informed consent was obtained from all patients for being included in the study.

## References

[1] O'Callaghan B , Bosch AM , Houlden H . An update on the genetics, clinical presentation, and pathomechanisms of human riboflavin transporter deficiency. J Inherit Metab Dis. 2019;42(4):598‐607. doi:10.1002/jimd.12053 30793323

[2] EFSA . Dietary reference values for nutrients summary report. EFSA J. 2017;14(12):e15121.

[3] Nutrition value (n.d.). www.voedingscentrum.nl/encyclopedie/vitamine-b2.aspx

[4] Bamji MS , Bhaskaram P , Jacob CM . Urinary riboflavin excretion and erythrocyte glutathione‐reductase activity in preschool‐children suffering from upper respiratory‐infections and measles. Ann Nutr Metab. 1987;31(3):191‐196. doi:10.1159/000177268 3592624

[5] Mosegaard S , Dipace G , Bross P , Carlsen J , Gregersen N , Olsen RKJ . Riboflavin deficiency‐implications for general human health and inborn errors of metabolism. Int J Mol Sci. 2020;21(11):3847. doi:10.3390/ijms21113847 32481712 PMC7312377

[6] Ho G , Yonezawa A , Masuda S , et al. Maternal riboflavin deficiency, resulting in transient neonatal‐onset glutaric aciduria type 2, is caused by a microdeletion in the riboflavin transporter gene GPR172B. Hum Mutat. 2011;32(1):E1976‐E1984. doi:10.1002/humu.21399 21089064

[7] Mosegaard S , Bruun GH , Flyvbjerg KF , et al. An intronic variation in SLC52A1 causes exon skipping and transient riboflavin‐responsive multiple acyl‐CoA dehydrogenation deficiency. Mol Genet Metab. 2017;122(4):182‐188. doi:10.1016/j.ymgme.2017.10.014 29122468

[8] Bashford JA , Chowdhury FA , Shaw CE . Remarkable motor recovery after riboflavin therapy in adult‐onset Brown‐Vialetto‐Van Laere syndrome. Pract Neurol. 2017;17(1):53‐56. doi:10.1136/practneurol-2016-001488 27777325 PMC5520257

[9] Chaya S , Zampoli M , Gray D , et al. The first case of riboflavin transporter deficiency in sub‐Saharan Africa. Semin Pediatr Neurol. 2018;26:10‐14. doi:10.1016/j.spen.2017.03.002 29961494

[10] Manole A , Jaunmuktane Z , Hargreaves I , et al. Clinical, pathological and functional characterization of riboflavin‐responsive neuropathy. Brain. 2017;140:2820‐2837. doi:10.1093/brain/awx231 29053833 PMC5808726

[11] Ciccolella M , Corti S , Catteruccia M , et al. Riboflavin transporter 3 involvement in infantile Brown‐Vialetto‐Van Laere disease: two novel mutations. J Med Genet. 2013;50(2):104‐107. doi:10.1136/jmedgenet-2012-101204 23243084

[12] Powers HJ . Riboflavin (vitamin B‐2) and health. Am J Clin Nutr. 2003;77(6):1352‐1360. 12791609 10.1093/ajcn/77.6.1352

[13] Jaeger B , Bosch AM . Clinical presentation and outcome of riboflavin transporter deficiency: mini review after five years of experience. J Inherit Metab Dis. 2016;39(4):559‐564. doi:10.1007/s10545-016-9924-2 26973221 PMC4920840

[14] Srour M , Putorti ML , Schwartzentruber J , et al. Mutations in riboflavin transporter present with severe sensory loss and deafness in childhood. Muscle Nerve. 2014;50(5):775‐779. doi:10.1002/mus.24224 24616084

[15] Allison T , Roncero I , Forsyth R , Coffman K , Pichon JL . Brown‐Vialetto‐Van Laere syndrome as a mimic of Neuroimmune disorders: 3 cases from the clinic and review of the literature. J Child Neurol. 2017;32(6):528‐532. doi:10.1177/0883073816689517 28116953

[16] Malafronte P , Clark HB , Castaneda‐Sanchez I , Raisanen J , Hatanpaa KJ . Brown‐Vialetto‐Van Laere syndrome: clinical and neuropathologic findings with immunohistochemistry for C20orf54 in three affected patients. Pediatr Dev Pathol. 2013;16(5):364‐371. doi:10.2350/13-02-1299-CR.1 23688382

[17] Bandettini Di Poggio M , Monti Bragadin M , Reni L , et al. Brown‐Vialetto‐Van Laere syndrome: clinical and neuroradiological findings of a genetically proven patient. Amyotroph Lateral Scler Frontotemporal Degener. 2014;15(1–2):141‐144. doi:10.3109/21678421.2013.837931 24079556

[18] Foley AR , Menezes MP , Pandraud A , et al. Treatable childhood neuronopathy caused by mutations in riboflavin transporter RFVT2. Brain. 2014;137(Pt 1):44‐56. doi:10.1093/brain/awt315 24253200 PMC3891447

[19] Brucher JM , Dom R , Lombaert A , Carton H . Progressive pontobulbar palsy with deafness: clinical and pathological study of two cases. Arch Neurol. 1981;38(3):186‐190. doi:10.1001/archneur.1981.00510030080012 6970563

[20] Anand G , Hasan N , Jayapal S , et al. Early use of high‐dose riboflavin in a case of Brown‐Vialetto‐Van Laere syndrome. Dev Med Child Neurol. 2012;54(2):187‐189. doi:10.1111/j.1469-8749.2011.04142.x 22098162

[21] Spagnoli C , Pitt MC , Rahman S , de Sousa C . Brown‐Vialetto‐van Laere syndrome: a riboflavin responsive neuronopathy of infancy with singular features. Eur J Paediatr Neurol. 2014;18(2):231‐234. doi:10.1016/j.ejpn.2013.09.006 24206674

[22] Alcorta A , Porta A , Tarrega A , Alvarez MD , Vaquero MP . Foods for plant‐based diets: challenges and innovations. Foods. 2021;10(2):293. doi:10.3390/foods10020293 33535684 PMC7912826

